# Azithromycin Promotes the Osteogenic Differentiation of Human Periodontal Ligament Stem Cells after Stimulation with TNF-*α*

**DOI:** 10.1155/2018/7961962

**Published:** 2018-10-31

**Authors:** Tingting Meng, Ying Zhou, Jingkun Li, Meilin Hu, Xiaomeng Li, Pingting Wang, Zhi Jia, Liyu Li, Dayong Liu

**Affiliations:** ^1^Department of Endodontics & Laboratory of Stem Cells and Endocrine Immunology, Tianjin Medical University School of Stomatology, Tianjin 300070, China; ^2^Department of Prosthodontics, Tianjin Medical University School of Stomatology, Tianjin 300070, China; ^3^Department of Intensive Care Unit, The Second Hospital of Tianjin Medical University, Tianjin 300211, China

## Abstract

**Background and Objective:**

This study investigated the effects and underlying mechanisms of azithromycin (AZM) treatment on the osteogenic differentiation of human periodontal ligament stem cells (PDLSCs) after their stimulation with TNF-*α in vitro. Methods*. PDLSCs were isolated from periodontal ligaments from extracted teeth, and MTS assay was used to evaluate whether AZM and TNF-*α* had toxic effects on PDLSCs viability and proliferation. After stimulating PDLSCs with TNF-*α* and AZM, we analyzed alkaline phosphatase staining, alkaline phosphatase activity, and alizarin red staining to detect osteogenic differentiation. Real-time quantitative polymerase chain reaction (RT-qPCR) analysis was performed to detect the mRNA expression of osteogenic-related genes, including *RUNX2*, *OCN*, and *BSP*. Western blotting was used to measure the NF-*κ*B signaling pathway proteins p65, phosphorylated p65, I*κ*B-*α*, phosphorylated I*κ*B-*α*, and *β*-catenin as well as the apoptosis-related proteins caspase-8 and caspase-3. Annexin V assay was used to detect PDLSCs apoptosis.

**Results:**

TNF-*α* stimulation of PDLSCs decreased alkaline phosphatase and alizarin red staining, alkaline phosphatase activity, and mRNA expression of *RUNX2*, *OCN*, and *BSP* in osteogenic-conditioned medium. AZM enhanced the osteogenic differentiation of PDLSCs that were stimulated with TNF-*α*. Western blot analysis showed that *β*-catenin, phosphorated p65, and phosphorylated I*κ*B-*α* protein expression decreased in PDLSCs treated with AZM. In addition, pretreatment of PDLSCs with AZM (10 *μ*g/ml, 20 *μ*g/ml) prevented TNF-*α*-induced apoptosis by decreasing caspase-8 and caspase-3 expression.

**Conclusions:**

Our results showed that AZM promotes PDLSCs osteogenic differentiation in an inflammatory microenvironment by inhibiting the WNT and NF-*κ*B signaling pathways and by suppressing TNF-*α*-induced apoptosis. This suggests that AZM has potential as a clinical therapeutic for periodontitis.

## 1. Introduction

Periodontitis is a chronic infectious disease of the periodontal supportive tissues, and it is the main cause of tooth loss in adults. Its pathological manifestations include gingival and periodontal ligament inflammatory infiltration, periodontal pocket formation, progressive attachment loss, and alveolar bone destruction [1]. Growing evidence demonstrates the correlation between periodontitis and systemic disorders such as diabetes, cardiovascular diseases, preterm birth, and low birth weight [2, 3]. A recent report identified periodontal disease as a risk factor for non-Hodgkin lymphoma and colorectal cancer [4]. Etiological evidence shows that periodontal pathogens in the dental biofilm under the gingival epithelium are necessary but insufficient for periodontitis development. Accumulating evidence shows that host susceptibility rather than bacterial plaque leads to periodontal destruction. Indeed, the host inflammatory response plays an essential role in the pathogenesis of periodontitis [5, 6].

Mesenchymal stem cells (MSCs) were first isolated from bone marrow and possess self-renewal, colony-forming unit, and immunomodulation properties. Notably, MSCs can differentiate into osteoblasts, adipocytes, chondrocytes, and neural cells [7]. MSCs play important roles in tissue hemostasis and in maintaining the balance between effective and regulative immune cells [8], and impaired MSCs in bone marrow or in local tissue may cause disease. We demonstrated previously that MSCs derived from the periodontal ligament tissues of patients with periodontitis showed impaired differentiation and immunomodulation that contributed to the development of periodontal tissue destruction [9–11]. Recent reports indicate the close relationship between impaired MSCs and autoimmune or inflammatory diseases [12]. Indeed, MSC transplantation is a successful therapeutic strategy for treating autoimmune diseases such as SLE [13]; Sjögren syndrome, autoimmune diabetes, and airway inflammation [14]; systemic sclerosis [15]; and periodontitis [16–18]. However, the mechanisms underlying MSC deficiency in periodontitis remain poorly defined and it is unclear how to restore MSC function and achieve periodontal tissue regeneration in an inflammatory microenvironment.

Azithromycin (AZM) is a clinically available macrolide antibiotic like erythromycin A and clarithromycin [19]. In addition to their antimicrobial activity, macrolides can modulate the immune response and inflammation with no effects on homeostatic immunity [20]. In epithelial and immune cells, low-dose macrolides inhibit the secretion of proinflammatory cytokines and chemokines, including IL-6, IL-8, and TNF-*α* [21, 22]. They also suppress interferon gamma production by memory T cells [23]. CSY0073, an AZM derivative that lacks antibiotic activity, improves the clinical scores of dextran sulfate sodium- (DSS-) induced experimental colitis and collagen-induced arthritis [24]. AZM is reported to be transported into inflamed tissues in the periodontium. After 3 days of daily administration of a single dose of AZM (500 mg), AZM can be detected for up to 6.5 days in the plasma, saliva, and inflamed periodontal tissues of human subjects [25]. Although there are no definitive, controlled clinical studies on the effects of AZM on periodontitis, AZM elicits clinical and microbiological improvement when used in conjunction with nonsurgical periodontal therapy [26–30]. Moreover, one study reported that AZM suppresses human osteoclast differentiation and bone resorption [31]. However, it remains unclear whether AZM affects osteoblasts or the osteogenesis of MSCs in an inflammatory microenvironment.

This study isolated human periodontal ligament stem cells (PDLSCs) and stimulated them with the proinflammatory cytokine TNF-*α in vitro*. Osteogenic differentiation and cell viability were determined in order to investigate the effects and underlying mechanisms of AZM on the osteogenic differentiation of PDLSCs in an inflammatory microenvironment. Our results showed that AZM promoted PDLSCs osteogenic differentiation after TNF-*α* stimulation by inhibiting the WNT and NF-*κ*B signaling pathways and by attenuating TNF-*α*-induced apoptosis.

## 2. Materials and Methods

### 2.1. Cell Culture

All researches involving human stem cells complied with the ISSCR “Guidelines for the Conduct of Human Embryonic Stem Cell Research.” PDLSCs were isolated from healthy volunteers who had no history of periodontal diseases and who had relatively healthy periodontiums. All of the experiments followed the guidelines of the Tianjin Medical University School of Stomatology. We obtained written informed consent from all volunteers prior to collecting their cells. PDLSCs were isolated, cultured, and identified as described previously [32]. Generally, the middle one-third of the periodontal ligament was extracted from the surface of the tooth root and then subjected to a gradient wash. Next, the chopped tissues were digested in a solution of 3 mg/ml collagenase type I plus 4 mg/ml dispase (Sigma-Aldrich, St. Louis, MO, USA) for 1 h at 37°C.

The PDLSCs from all of the volunteers were pooled. A single-cell suspension was prepared by passing the cells through a 70 *μ*m strainer (Falcon, BD Labware, Franklin Lakes, NJ, USA), and PDLCSs were plated in complete *α*-MEM (HyClone, Logan, UT, USA) plus 20% FBS (Gibco, Carlsbad, CA, USA), 100 U/ml penicillin, and 100 *μ*g/ml streptomycin (Invitrogen, Carlsbad, CA, USA). The cells were cultured at 37°C in 5% carbon dioxide, and the culture medium was changed every 3 days. Passages 3–6 were used for the experiments. A total of 15 volunteers, aged 18 to 23 years old, provided informed written consent. PDLSCs were identified by flow cytometry using antibodies against STRO-1, CD90, CD45, and CD146. The details are described in the Supplementary Materials and Methods (available here).

### 2.2. MTS Assay

Cell viability was measured using an MTS assay (Promega, Madison, WI, USA). PDLSCs were seeded in 96-well plates at a density of 3 × 10^3^ cells/well and cultured to approximately 80% confluence. TNF-*α* (20 ng/ml, 100 ng/ml) and AZM (1 *μ*g/ml, 10 *μ*g/ml, and 20 *μ*g/ml) were added. The cells were cultured in osteogenic medium for 48 h at 37°C and then incubated for 3 h with MTS. The OD_490_ was measured using a microplate reader. The experiments, which had 7 replicates, were repeated at least 3 times.

### 2.3. Alizarin Red Staining and Quantitative Calcium Analysis

PDLSCs were fixed in 70% ethanol for 1 h and washed with deionized water. We added 40 mM alizarin red staining solution (pH 4.2) into the 6-well plates, incubated the cells at room temperature for 10 min, washed the cells with deionized water 5 times, viewed them under a microscope, and captured the images. For quantitative calcium analysis, the cells were treated with 10% cetylpyridinium chloride solution (Sigma-Aldrich) for 30 min at room temperature. The OD_562_ was used to quantify the degree of mineralization and calcium quantitative analysis for alizarin red staining was normalized to the total protein content before calculation. The experiments were repeated at least 3 times.

### 2.4. Alkaline Phosphatase Staining

PDLSCs were seeded in 6-well plates. In addition to the control conditions, there were 3 experimental conditions: 100 ng/ml TNF-*α*, 100 ng/ml TNF-*α* plus 10 *μ*g/ml AZM, and 100 ng/ml TNF-*α* plus 20 *μ*g/ml AZM. We examined osteogenesis at 7 days and acquired images. The alkaline phosphatase (ALP) activity assay is described in the Supplementary Materials and Methods.

### 2.5. Quantitative Real-Time PCR

Total RNA was isolated from PDLSCs using TRIzol reagent (Life Technologies, Carlsbad, CA, USA). We used oligo (dT) primers and reverse transcriptase to amplify cDNA according to the manufacturer's protocol (Invitrogen). RT-qPCR was performed using the SYBR Green PCR kit (Qiagen, Düsseldorf, Germany). Each reaction was repeated at least three times. Supplementary Table 1 shows the primers for specific genes.

### 2.6. Western Blot Analysis

Total proteins were extracted from PDLSCs by lysing the cells in RIPA buffer (10 mM Tris-HCl, 1 mM EDTA, 1% sodium dodecyl sulfate (SDS), 1% NP-40, 1 : 100 proteinase inhibitor cocktail, 50 mM *β*-glycerophosphate, and 50 mM sodium fluoride) and 1% PMSF. The proteins were separated on 10% and 12% SDS polyacrylamide gels and then electrotransferred to polyvinylidene fluoride (PVDF) membranes for 2 h at 300 mA. The membranes were incubated overnight with primary antibodies at 4°C. Primary monoclonal antibodies directed against the following were used in this study: phosphorylated p65, p65, phosphorylated I*κ*B-*α*, I*κ*B-*α*, the housekeeping protein glyceraldehyde phosphate dehydrogenase (GAPDH, Abcam, Cambridge, MA, USA), caspase-3, and caspase-8 (Cell Signaling Technology Inc.) Blots were then incubated with the secondary antibody (peroxidase-conjugated goat anti-rabbit; 1 : 1000, Abcam) for 2 h at room temperature. GAPDH was used as the internal control. Each experiment had three replicates and was repeated at least three times.

### 2.7. Cell Apoptosis Assay

Cells were seeded at a density of 2 × 10^3^/cm^2^. After treatment with 100 ng/ml TNF-*α* or 10 *μ*g/ml AZM plus 20 *μ*g/ml AZM for 24 h, PDLSCs were stained with annexin V-fluorescein isothiocyanate (FITC) and counterstained with propidium iodide (PI). The eBioscience™ annexin V-FITC Apoptosis Detection Kit (Life Technologies) was used. Briefly, cells were washed twice with phosphate-buffered saline (PBS) and then stained with 200 *μ*l binding buffer (1x) and 5 *μ*l annexin V-FITC for 10 min at room temperature in the dark. Finally, 10 *μ*l of PI in 1x binding buffer was added to the cells for 5 minutes. The cells were analyzed using a fluorescence microscope.

### 2.8. Annexin V Apoptosis Assay

We washed 1 × 10^5^ cells twice with PBS followed by centrifugation at 4°C at 2000 rpm for 5 minutes to collect cell pellets. The cell pellets were resuspended in 200 *μ*l binding buffer (1x) and stained with 5 *μ*l of annexin V-FITC for 10 min at room temperature in the dark, and then 10 *μ*l PI in 1x binding buffer was added to the cell suspension. The cells were analyzed by fluorescence-activated cell sorting (FACs). Each experiment was performed in triplicate.

### 2.9. Statistical Analysis

The data are reported as means ± SD. We used one-way ANOVA for statistical analysis, and a *P* value < 0.05 was considered significant.

## 3. Results

### 3.1. TNF-*α* and AZM at Experimental Levels Had No Toxic Effects on PDLSC Viability or Proliferation

PDLSCs have an elongated spindle morphology (Figure S1). Flow cytometry results for biomarkers are shown in Figure S2. To investigate whether different concentrations of TNF-*α* and AZM affected cell proliferation and viability, we used MTS assay to compare the viability of PDLSCs cultured in osteogenic conditions versus PDLSCs treated with TNF-*α* and AZM (Figure S3). TNF-*α* was used at two concentrations (20 ng/ml, 100 ng/ml) and AZM at three concentrations (1 *μ*g/ml, 10 *μ*g/ml, and 20 *μ*g/ml). TNF-*α* treatment alone tended to reduce the number of viable cells, although this reduction was not significant. Based on these results, we chose to use 20 ng/ml and 100 ng/ml TNF-*α* and 10 *μ*g/ml and 20 *μ*g/ml AZM as working concentrations for the subsequent experiments.

### 3.2. Effects of AZM on the Osteogenic Differentiation of PDLSCs

**Figure 1:**
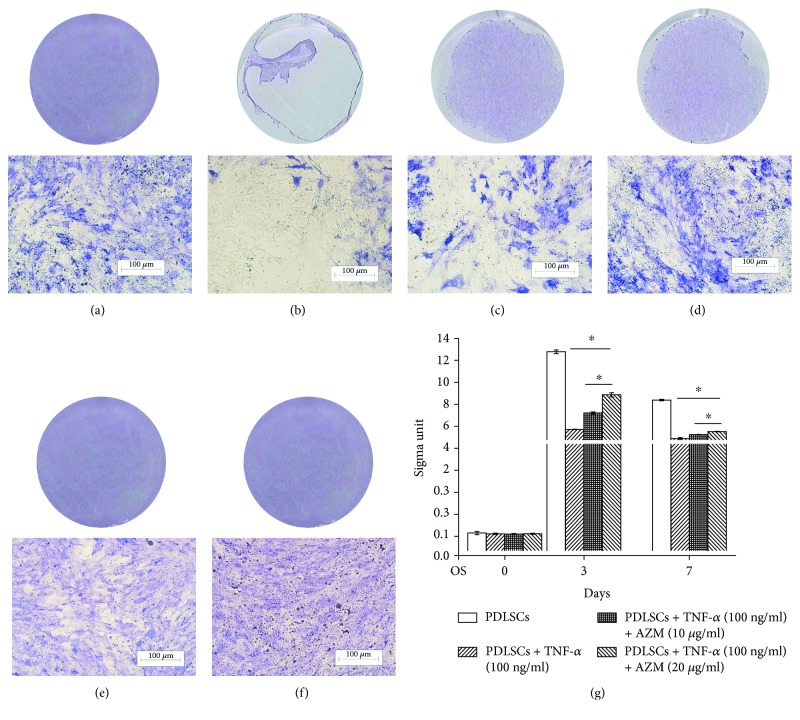
Analysis of alkaline phosphatase staining and alkaline phosphatase activity in human PDLSCs after treatment with AZM. (a–f) PDLSCs were cultured in osteogenic medium for 7 days. (a) Control PDLSCs cultured without any additions. (b) PDLSCs treated with TNF-*α* (100 ng/ml). (c) PDLSCs treated with TNF-*α* (100 ng/ml) and AZM (10 *μ*g/ml). (d) PDLSCs treated with TNF-*α* (100 ng/ml) and AZM (20 *μ*g/ml). (e) PDLSCs treated with AZM (10 *μ*g/ml). (f) PDLSCs treated with AZM (20 *μ*g/ml). (g) Alkaline phosphatase activity analysis. PDLSCs were induced to form osteoblasts for 0, 3, or 7 days. The results showed that AZM promoted the ability of PDLSCs to undergo osteogenesis differentiation. ^∗^ *P* < 0.05 indicates significant differences. Data are presented as means ± SD.

**Figure 2:**
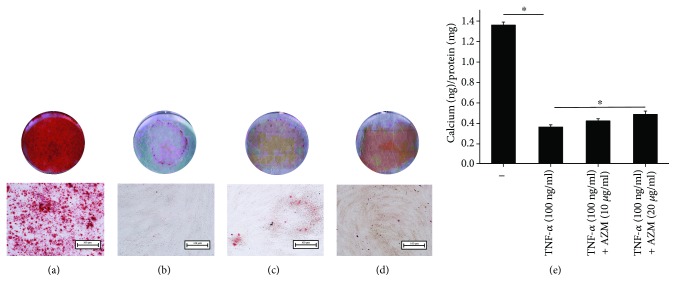
Alizarin red staining of human PDLSCs cultured in osteogenic media for 7 days. (a–d) PDLSCs cultured in osteogenic medium for 7 days. (a) Control PDLSCs cultured without any additions. (b) PDLSCs treated with TNF-*α* (100 ng/ml). (c) PDLSCs treated with TNF-*α* (100 ng/ml) and AZM (10 *μ*g/ml). (d) PDLSCs treated with TNF-*α* (100 ng/ml) and AZM (20 *μ*g/ml). (e) Detection of the calcium ion concentration and the calcium quantitative analysis for alizarin red staining were normalized to the total protein content before calculation. Increasing the AZM concentration significantly changed the number of calcium nodules. The results showed that AZM promoted the osteogenic differentiation of PDLSCs. ^∗^ *P* < 0.05 indicates significant differences. Data are presented as means ± SD.

To investigate the effects of AZM on the osteogenic differentiation of PDLSCs, cells were cultured in osteogenic medium for 7 days. Experimental PDLSCs were treated with TNF-*α* (100 ng/ml) and AZM (10 *μ*g/ml, 20 *μ*g/ml). The ALP staining results (Figure 1) and alizarin red staining results (Figure 2) showed that AZM can restore the ability of PDLSCs to undergo osteogenic differentiation after the cells are impaired by TNF-*α* (100 ng/ml). Compared to control cells that underwent osteogenic induction, TNF-*α* treatment decreased staining and calcium nodule formation (Figure 2). Notably, TNF-*α* is a proinflammatory cytokine that contributes to bone loss in many different diseases. Until now, the mechanisms by which TNF-*α* inhibits osteogenic differentiation have been unclear and have been thought to be complex. In accordance with previous results, TNF-*α* reduced osteogenic differentiation and our data suggested that it decreased the number of calcium nodules that were formed as well (Figure 2(e)). Cotreatment of PDLSCs with TNF-*α* (100 ng/ml) and AZM (20 *μ*g/ml) rescued the cells' ability to undergo osteogenesis compared with the TNF-*α* group, even though osteogenesis was lower than that for control cells. The higher the AZM concentration, the deeper the blue or red staining is. This suggests that AZM has a positive role in human PDLSC osteogenic differentiation, since cells underwent osteogenesis when they were cultured in the absence or presence of TNF-*α* and AZM for 0, 3, or 7 days.

Similar to the ALP staining and alizarin red staining results, analysis of ALP activity demonstrated that AZM caused PDLSCs to regain their osteogenic ability (Figure 1(g)). Remarkably, the cells that were treated with TNF-*α* alone clearly had fewer cells (Figures 1(b) and 2(b)). As the AZM concentration increased, the number of cells increased as well.

**Figure 3:**
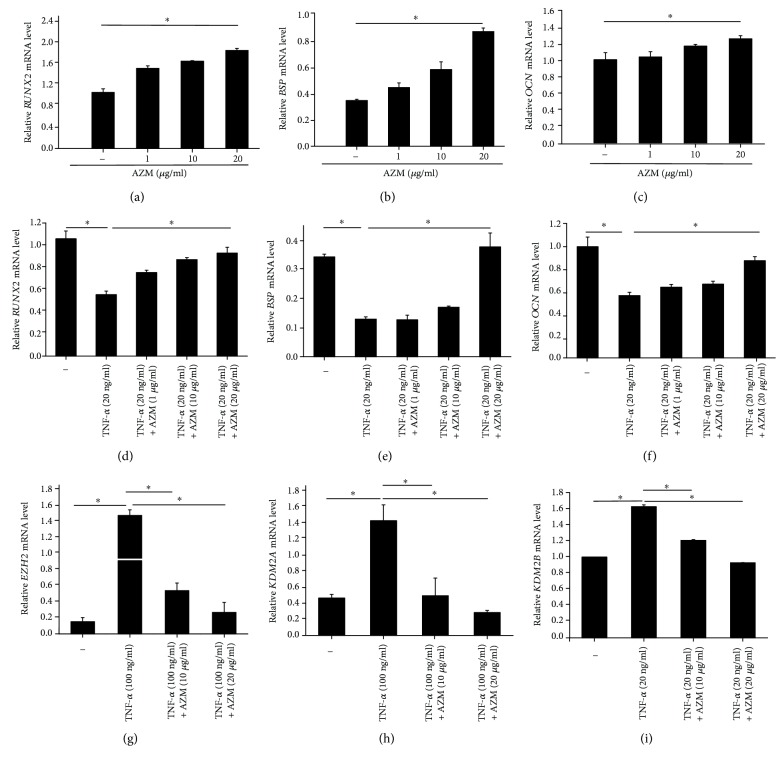
RT-qPCR analysis showed that AZM promotes the osteogenic differentiation of human PDLSCs and impacts the mRNA levels of epigenetic-related genes. Quantitative real-time PCR analysis of *RUNX2*, *BSP*, *OCN*, *KDM2A*, *KDM2B*, and *EZH2*. PDLSCs were treated with TNF-*α* and AZM as indicated. The top three images show the mRNA levels of (a) *RUNX2*, (b) *BSP*, and (c) *OCN* in cells treated with 1 *μ*g/ml, 10 *μ*g/ml, and 20 *μ*g/ml AZM, respectively. The middle three images show the mRNA levels of (d) *RUNX2*, (e) *BSP*, and (f) *OCN* treated with 20 ng/ml TNF-*α* and 1 *μ*g/ml, 10 *μ*g/ml, or 20 *μ*g/ml AZM. The mRNA levels of (g) *EZH2*, (h) *KDM2A*, and (i) *KDM2B* are shown in the bottom three images. PDLSCs were treated with 20 ng/ml TNF-*α* and 10 *μ*g/ml AZM or with 20 ng/ml TNF-*α* and 20 *μ*g/ml AZM. ^∗^ *P* < 0.05 indicates significant differences. The data are presented as means ± SD.

We speculated that AZM could promote osteogenesis and could partially restore PDLSC osteogenic capacity in an inflammatory microenvironment. To verify this, we assessed the mRNA expression of the osteogenic differentiation markers *OCN*, *BSP*, and *RUNX2* by real-time PCR (Figure 3). We found that AZM treatment promoted PDLSCs osteogenic differentiation and the mRNA expression of these genes in a dose-dependent manner (Figure 3(a)–3(f)). When cells were exposed to an inflammatory microenvironment (i.e., treated with TNF-*α*), the mRNA levels of *OCN*, *BSP*, and *RUNX2* were lower than those in control (*P* < 0.05). However, cotreatment with AZM restored the mRNA expression levels (Figure 3(a)–3(f)). The mRNA expression levels of *KDM2A*, *KDM2B*, and *EZH2* were higher in TNF-*α*-treated cells compared to control cells, and AZM mitigated this effect (Figure 3(g)–3(i)).

### 3.3. AZM Rescued the Osteogenic Potential of PDLSCs through the WNT and NF-*κ*B Signaling Pathways

**Figure 4:**
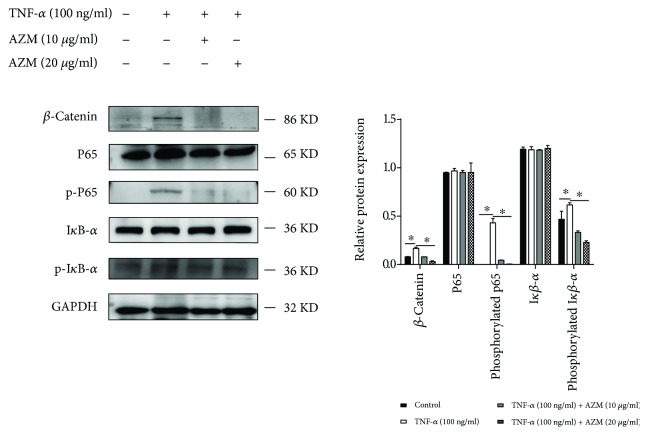
AZM restored the osteogenic potential of human PDLSCs through the WNT and NF-*κ*B signaling pathways. PDLSCs were cultured in osteogenic medium and treated with 100 ng/ml TNF-*α* and AZM (10 *μ*g/ml, 20 *μ*g/ml) for 7 days. The protein levels of p65, phosphorylated p65, *β*-catenin, I*κ*B-*α*, and phosphorylated I*κ*B-*α* were detected by Western blot analysis. ^∗^ *P* < 0.05 indicates significant differences. Data are presented as means ± SD.

In an inflammatory environment, NF-*κ*B plays a vital role in the osteogenic differentiation of PDLSCs [33]. We next asked whether TNF-*α*-induced osteogenic inhibition could be partially reversed in the presence of AZM through the suppression of NF-*κ*B signaling. Accordingly, we used Western blotting to analyze the expression of p65, phosphorylated p65, I*κ*B-*α*, and phosphorylated I*κ*B-*α* (Figure 4). After 7 days of osteogenic differentiation, TNF-*α* promoted the expression of phosphorylated p65 and phosphorylated I*κ*B-*α* in PDLSCs compared with control. However, when PDLSCs were treated with both 100 ng/ml TNF-*α* and 20 *μ*g/ml AZM, the levels of phosphorylated p65 and phosphorylated I*κ*B-*α* were lower than those in cells treated with TNF-*α* alone. We also detected the levels of p65 and I*κ*B*α*. The protein level is shown in Figure 4.

Consistent with the ALP and alizarin staining results, TNF-*α* inhibited PDLSC osteogenic differentiation, while AZM partially reversed this effect and promoted PDLSC osteogenic differentiation. Phosphorylated p65 reflects the activation of the NF-*κ*B signaling pathway. Thus, the results showed that AZM promoted osteogenic differentiation by suppressing the NF-*κ*B signaling pathway. We then investigated whether WNT signaling plays a role in this process. We found that *β*-catenin expression increased after cells were treated with TNF-*α*. These results suggested that AZM promoted the osteogenic differentiation of PDLSCs in an inflammatory microenvironment by inhibiting the activation of the WNT and NF-*κ*B signaling pathways.

### 3.4. AZM Promotes PDLSC Osteogenic Differentiation by Suppressing TNF-*α*-Induced Apoptosis

**Figure 5:**
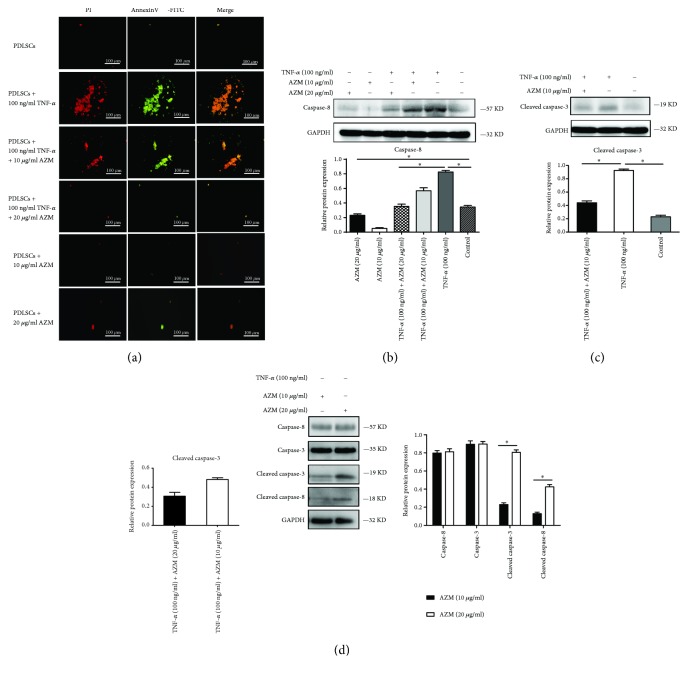
Immunocytochemical staining and the expression levels of the apoptosis proteins caspase-3 and caspase-8 in human PDLSCs. (a) Immunofluorescence staining. PDLSCs were incubated in osteogenic medium with or without TNF-*α* and AZM as indicated for 24 h. (b–d) The expression levels of caspase-3,caspase-8,cleaved caspase-3, and cleaved caspase-8 were detected by Western blot analysis. The results showed that TNF-*α* induced cell apoptosis and that AZM treatment prevented PDLSCs from undergoing TNF-*α*-induced apoptosis. AZM alone at 10 or 20 *μ*g/ml had no effect on apoptosis. ^∗^ *P* < 0.05 indicates significant differences. Data are presented as means ± SD.

TNF-*α* is a strong apoptosis-promoting factor. There were some indications that AZM might repress the TNF-*α*-induced apoptosis of PDLSCs, since ALP staining showed that TNF-*α* treatment alone reduced the number of viable cells and that cotreatment with TNF-*α* plus AZM mitigated this effect (Figures 1 and 2). To investigate the mechanism underlying this phenomenon, we tested whether AZM rescued PDLSC osteogenesis by suppressing TNF-*α*-induced apoptosis. PDLSCs were seeded at a density of 2 × 10^3^/cm^2^ in 6-well plates with or without TNF-*α* (100 ng/ml) and AZM (10 *μ*g/ml, 20 *μ*g/ml) for 24 hours. Notably, 100 ng/ml TNF-*α* promoted PDLSCs apoptosis and AZM mitigated this process. In addition, AZM did not promote PDLSCs apoptosis. PI staining and FITC staining were used to follow apoptosis in PDLSCs undergoing osteogenic differentiation and showed that 10 *μ*g/ml or 20 *μ*g/ml AZM had no effect on apoptosis (Figure 5(a)).

In the caspase activation process, the caspase prodomain is cleaved and caspase proteins form a heterotetrameric enzyme in response to proteolytic activation. Next, protein downstream of caspase is activated, resulting in apoptosis [34, 35]. Caspase-8 is an initiator caspase, and caspase-3 is an effector caspase. To further investigate the mechanisms underlying the effects of AZM, we determined the protein levels of caspase-3, caspase-8, cleaved caspase-3, and cleaved caspase-8. The results demonstrated that after PDLSCs underwent osteogenic differentiation for 7 days, the protein levels of caspase-8 and cleaved caspase 3 were high in cells treated with TNF-*α* alone and lower when AZM was added (Figures 5(b) and 5(c)). PDLSCs treated with 10 *μ*g/ml or 20 *μ*g/ml AZM (Figure 5(d)) showed no differences in the protein levels of caspase-3 and caspase-8.

**Figure 6:**
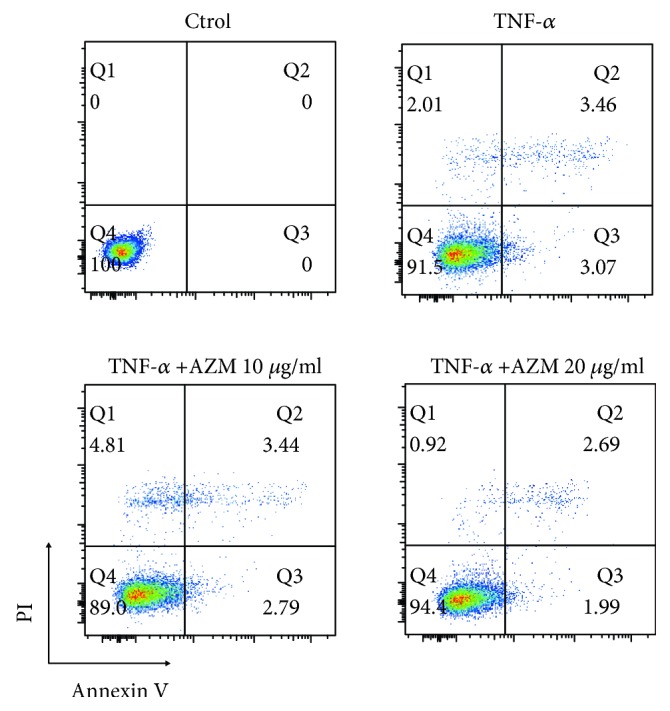
Detection of apoptosis in human PDLSCs by flow cytometry. PDLSCs were cultured in standard medium and treated with 100 ng/ml TNF-*α* and 10 *μ*g/ml or 20 *μ*g/ml AZM as indicated for 24 h. Apoptosis was detected using annexin V apoptosis assay.

Compared with levels in cells treated with 10 *μ*g/ml AZM, the levels of cleaved caspase-3 and cleaved caspase-8 were higher in cells treated with 20 *μ*g/ml AZM. It is possible that AZM promotes PDLSC differentiation. A more favorable cellular state can increase cell proliferation, although the MTS results showed no statistically significant differences in the proliferation of cells treated with AZM (Figure S3). Compared with cells treated with 10 *μ*g/ml AZM, cells treated with 20 *μ*g/ml AZM showed a slightly increased cell number. The level of apoptosis in cells treated with AZM was lower than in that cells treated with TNF-*α* and control cells (Figures 5(b) and 5(d)). AZM inhibited apoptosis in a dose-dependent manner (Figures 5(b) and 5(d)). To confirm if AZM promotes human PDLSC osteogenesis differentiation associated with the suppression of TNF-*α*-induced apoptosis, PDLSCs were cultured in basal medium and then cultured with or without TNF-*α* (100 ng/ml) and AZM (10 *μ*g/ml, 20 *μ*g/ml) for 24 hours. Annexin V-positive cells were detected by flow cytometry analysis. Moderate levels of TNF-*α* can promote apoptosis in PDLSCs, but AZM mitigated this effect. Compared with PDLSCs treated with TNF-*α* alone (Figure 6), the apoptosis level decreased in the presence of AZM. Our data thus showed that AZM can block TNF-*α*-induced apoptosis.

Taken together, these data demonstrate that AZM promotes the osteogenic differentiation of PDLSCs by suppressing TNF-*α*-induced apoptosis.

## 4. Discussion

Periodontitis is a complex progressive inflammatory disease that is more prevalent in adults but also occurs in children and adolescents. Notably, periodontitis can lead to alveolar bone loss and systemic inflammation. Dysbiosis of the dental plaque, which interacts with the host immune defense, initiates periodontitis. Because the underlying mechanism is complex, it is challenging to repair bone loss and improve the deep periodontal pocket to achieve a satisfactory end result [9]. Bartold et al. demonstrated that dental plaque is essential but insufficient for periodontitis [4, 5, 36, 37]. AZM has anti-inflammatory properties and it is reported by several groups [38, 39]. Here, we found that AZM can reverse bone loss and suppress PDLSC apoptosis. PDLSCs that can differentiate into osteoblasts show great potential for treating patients with periodontitis.

TNF-*α* is a strong apoptosis inducer and a proinflammatory cytokine that contributes to bone loss in local and systemic inflammatory bone diseases [40]. TNF-*α* inhibits the expression of the osteogenic-related gene *Runx2* in two ways. First, it suppresses *Runx2* gene expression. Second, it promotes *Runx2* degradation [41]. Our data provide evidence that AZM promotes PDLSCs osteogenic differentiation in an inflammatory microenvironment.

This study had four major findings. First, PDLSCs osteogenesis was strikingly inhibited by TNF-*α* and clearly enhanced by AZM. Second, Western blot analysis showed that TNF-*α* increased the expression of phosphorylated p65, phosphorylated I*κ*B-*α*, and *β*-catenin. In contrast, the levels of these proteins were inhibited by AZM in a concentration-dependent manner. Third, stimulation with TNF-*α* activated the cleaved caspase-3 protein and AZM reversed the TNF-*α*-induced apoptosis of PDLSCs. Fourth, flow cytometry analysis showed that moderate concentrations of TNF-*α* promoted PDLSC apoptosis and that AZM mitigated this process. Our finding that AZM can inhibit the apoptosis of PDLSCs is consistent with the work of Mizunoe et al. [42] and Stamatiou et al. [43].

Our data shed light on the mechanisms by which AZM promotes osteogenesis. When trimeric TNF-*α* binds to TNFR1, the TNFR1-associated death domain protein (TRADD) is recruited to TNFR1. TRADD then acts as a bridge to recruit other apoptosis-related proteins, such as receptor-interacting protein (RIP), TNF receptor-associated factor 2 (TRAF2), and the Fas-associated death domain protein (FADD). Next, the integration of TRAF2 and RIP leads to the recruitment of the IKK complex. Intriguingly, phosphorylated-I*κ*B*α* is then degraded and this activates the NF-*κ*B signaling pathway and mediates cell apoptosis. TNF-*α* can trigger cell apoptosis in another way, that is, via the caspase pathway. This pathway involves FADD, caspase-8, and caspase-3. Caspase-3 activation allows it to cleave related proteins and results in cell death [34]. AZM blocks bone loss induced by TNF-*α* in two ways. First, it suppresses the activation of NF-*κ*B signaling, and second, it inhibits the cleavage of caspase family proteins.

Increasing evidence shows that the WNT pathway plays an important role in bone metabolism. There are two WNT signaling pathways: the canonical pathway, termed the WNT/*β*-catenin pathway, and the noncanonical WNT/Ca^2+^ pathway. The activation state of *β*-catenin is central in the WNT/*β*-catenin pathway. When WNT proteins bind to Frizzled receptors, *β*-catenin is activated and accumulates in the cytoplasm. Stable *β*-catenin is transported into the nucleus and mediates the transcription of downstream genes ([44], Huang, and [45–47]). Notably, high expression of *β*-catenin decreased the mRNA expression of *Runx2*, *COL1*, and *OCN* in PDLSCs extracted from an inflammatory microenvironment. Some researchers asserted that high *β*-catenin expression decreases osteogenesis via the noncanonical pathway, while others considered this to occur via the canonical pathway [47, 48]. Because we examined the protein expression of *β*-catenin, we do not know which pathway AZM inhibits and additional experiments are needed to determine the precise mechanism.

Epigenetic regulation of gene expression is heritable and reversible. The DNA sequence is not altered in epigenetics; rather, there is methylation of lysine or arginine residues in the histone tails. The methylated lysine residues are considered epigenetic signals that may be related to gene activation, as for methylation at H3K4 and H3K36, or to gene repression, as for methylation at H3K9 and H3K27 [49, 50]. The histone lysine demethylases (KDMs) KDM2A and KDM2B demethylate H3K4me3 and H3K36me1/2 [50]. KDM2B plays an important role in BCOR mutation-associated diseases [51]. Moreover, the interactions between KDM2A and BCOR can inhibit osteogenesis by suppressing epiregulin (EREG) gene transcription, which is required for the expression of osterix (OSX) and distal-less homeobox 5 (DLX5) [52]. Our results are consistent with these reports.

KDM2B is a component of the noncanonical PRC1 (polycomb repressive complex 1), and it recruits Ring1B and Nspc1 to promote H2AK119 monoubiquitylation [53]. The recruited Ring1B may interact with RNA polymerase II (RNAPII), leading to a bivalent state [54]. KDM2B localizes to regions where H3K36me2 levels are low. TNF-*α* stimulation promotes the removal of the dimethyl markers at H3K36 and inhibits osteogenic-related gene transcription. EZH2, a member of PRC2 (polycomb repressive complex 2), is a type of histone lysine methyltransferase (KMT). EZH2 mainly catalyzes H3K27 trimethyl markers. The canonical PRC1 complex is recruited to the appropriate locations by PRC2, which can recognize H3K27me3 [55]. EZH2 has been known for decades to be a negative mediator of MSC osteogenesis, which is in accordance with our findings. AZM may promote osteogenesis through three ways. First, it can block PRC1 binding to the H2AK119ub promoter and then decrease the level of H2AK119ub. Second, it can increase the level of H3K36me2 and then promote gene transcription. Third, it can decrease the level of H3K27me3 and reduce the recruitment of PRC2, which can inhibit transcription inhibition and promote the expression of downstream genes.

Periodontal diseases contribute to the formation of a complex inflammatory microenvironment. This study showed that AZM has potential as a new drug for treating periodontal diseases. Although AZM cannot completely reverse bone loss, it is likely to be helpful to have some insights into the putative effects of AZM on periodontal diseases. There may be additional mechanisms involved that we did not explore here. In a TNF-*α*-induced inflammatory microenvironment, we detected the expression of osteoblast-specific genes and cell apoptosis *in vitro*, and we concluded that AZM promotes the osteogenic differentiation of PDLSCs in an inflammatory microenvironment by inhibiting the WNT and NF-*κ*B signaling pathways and the process associated with suppression of TNF-*α*-induced apoptosis. This study has some limitations. In particular, this was an *in vitro* study; animal studies were not conducted. Further experiments focusing on tissue regeneration are needed to better model the environment in humans.

In summary, our study suggested that AZM has potential as a new drug to treat periodontitis diseases and offered some insights into AZM and epigenetics. Further experiments are needed to investigate AZM as a therapeutic drug for periodontitis and bone tissue regeneration.

## 5. Conclusion

Our results showed that AZM promotes PDLSCs osteogenic differentiation in response to TNF-*α* stimulation by inhibiting the WNT and NF-*κ*B signaling pathways and by attenuating TNF-*α*-induced apoptosis.

## Data Availability

The data used to support the findings of this study are available from the corresponding author upon request.

## Abbreviations

BSP: Bone sialoprotein
EZH2: Enhancer of zeste homolog 2
FBS: Fetal bovine serum
FITC: Fluorescein isothiocyanate
KDM2A: Lysine-specific demethylase 2A
KDM2B: Lysine-specific demethylase 2B
OCN: Osteocalcin
PI: Propidium iodide
Runx2: Runt-related transcription factor 2.

## Supplementary Materials

Supplementary Materials The experimental section of alkaline phosphatase (ALP) activity assay and flow cytometric analysis were in the supplementary materials, and primers for specific genes sequences and the supplementary figures were also included. Supplementary Table 1: primers for gene sequences. Fig. S1: the image of PDLSCs under a 10x microscope. PDLSCs have the elongated spindle morphology. Fig. S2: PDLSCs cultured in normal medium. Flow cytometric analysis of PDLSCs showed positive expression of cell markers STRO-1, CD90, and CD146 and negative results for CD45. Fig. S3: appropriate TNF-*α* and azithromycin have no overt toxic effect on cell viability and proliferation of PDLSCs. Cell viability was measured using MTS assay; there are no significant difference between the 12 groups.
