# Supplementary material for: Azithromycin Promotes the Osteogenic Differentiation of Human Periodontal Ligament Stem Cells after Stimulation with TNF-α
Source: Stem Cells Int. 2018 Oct 31;2018:7961962. doi: 10.1155/2018/7961962 (PMC6234456; doi:10.1155/2018/7961962)
Supplement: Supplementary Materials — The experimental section of alkaline phosphatase (ALP) activity assay and flow cytometric analysis were in the supplementary materials, and primers for specific genes sequences and the supplementary figures were also included. Supplementary Table 1: primers for gene sequences. Fig. S1: the image of PDLSCs under a 10x microscope. PDLSCs have the elongated spindle morphology. Fig. S2: PDLSCs cultured in normal medium. Flow cytometric analysis of PDLSCs showed positive expression of cell markers STRO-1, CD90, and CD146 and negative results for CD45. Fig. S3: appropriate TNF-α and azithromycin have no overt toxic effect on cell viability and proliferation of PDLSCs. Cell viability was measured using MTS assay; there are no significant difference between the 12 groups. [file 7961962.f1.doc]

**Azithromycin Promotes the Osteogenic Differentiation of Human Periodontal Ligament Stem Cells After Stimulation with TNF-α**

Tingting Meng^1#^, Ying zhou^1#^, Jingkun Li^1^, Meilin Hu^1^, Xiaomeng Li^2^, Pingting Wang^1^, Zhi Jia^1^ , Liyu Li^3*^ , Dayong Liu^1*^

1. Department of Endodontics & Laboratory of Stem Cells and Endocrine Immunology, Tianjin Medical University School of Stomatology, Tianjin, 300070, China.
2. Department of Prosthodontics, Tianjin Medical University School of Stomatology, Tianjin, 300070, China.
3. Department of Intensive Care Unit, The Second Hospital of Tianjin Medical University, Tianjin 300211, China

* Corresponding author, 1. Dr. Dayong Liu, Department of Endodontics & Laboratory of Stem Cells and Endocrine Immunology, Tianjin Medical University School of Stomatology. Address, 12 Qixiangtai Road, Tianjin, 300070, China. Tel./Fax +86 22 2333 2095, Email: [dyliuperio@tmu.edu.cn](mailto:dyliuperio@tmu.edu.cn) or

2. Dr. Liyu Li, Department of Intensive Care Unit, The Second Hospital of Tianjin Medical University, Tianjin 300211, China. Tel./Fax +86 2283336825,E-mail: [tjydlly@126.com](mailto:tjydlly@126.com).

# These authors contribute equally to this work.

**Supplementary Materials**

**Supplementary materials and methods**

**Alkaline phosphatase (ALP) activity assay**

Cells were seeded into 6-well plates and cultured in osteogenic-inducing medium for 0days,3days and 7days.The ALP activity assay was performed according to the protocol of the ALP activity kit (Sigma−Aldrich, USA).We used standard samples to prepare the Standard curves, and then calculate the signal strength according to the standard samples.

**Flow cytometric analysis**

2.5 × 10^5^ third-passage PDLSCs were placed in the 1.5 ml Eppendorf tubes and washed with PBS twice. Next, cells were fixed with 4 % paraformaldehyde at room temperature for 15 minutes. Primary anti-STRO-1antibodies were added to the tubes and incubated at room temperature for 1 hour. The samples were washed with PBS and then secondary antibodies IgM were added to the tubes in the dark for 30 minutes. Fluorescein isothiocyanate (FITC)-conjugated or phycoerythrin (PE)-conjugated anti-CD146, anti-CD45, anti-CD90 antibodies were added to PDLSCs samples and incubated at room temperature in the dark for 30 minutes. The percentages of cells positively stained with STRO-1, CD146 and CD90 were assessed with fluorescence activated cell sorting, Each sample was performed in triplicate.

**Supplementary Table 1**

Primers for genes sequences

| **Gene Symbol** | **PRIMER SEQUENCES (5’—3’)** |
| --- | --- |
| *OCN*-F  *OCN*-R  *BSP*-F  *BSP*-R  *RUNX2* -F  *RUNX2-*R  *KDM2A-F*  *KDM2A-R*  *EZH2-F*  *EZH2-R*  *KDM2B-F*  *KDM2B-R*  *GAPDH*-F  *GAPDH*-R | 5'- CCTCACACTCCTCGCCCTATT -3'  5'- CCCTCCTGCTTGGACACAAA-3'  5'- AAACGAAGAAAGCGAAGCAGAA-3'  5'- GCTGCCGTTGCCGTTTT-3'  5'--ACCAGATGG GACTGTGGTTACT-3’  5'- GGATTAAAAGGACTTGGTGCAG-3’  5'-CGGATAGTTGAGAAAGCCAAGATCCG-3’  5'-CTCTTTGGTGGGCCTCTGTAGC-3’  5'- TCCTCCTGAATGTACCCCCA-3’  5'- TGCATGAAAAGGATGTAGGAAGC-3’  5'- GGACGCAAGCGGCTCAAAC-3’  5'- AGTTCGTCCTCGGGTTCCTG-3’GG  5'-CGGACCAATACGACCAAATCCG-3’  5'-AGCCACATCGCTCAGACACC-3’ |

**Supplement figure legends**

Fig. S1. The image of PDLSCs under a 10x microscope. PDLSCs have the elongated spindle morphology.

Fig. S2. PDLSCs cultured in normal medium. Flow cytometric analysis of PDLSCs showed positive expression of cell markers STRO -1, CD90 and CD146, and negative results for CD45.

Fig. S3. Appropriate TNF-ɑ and Azithromycin have no overt toxic effect on cell viability and proliferation of PDLSCs. Cell viability was measured using an MTS assay, there are no significant difference between the 12 groups.
